# Quantitative Neuromuscular Monitoring in General Anesthesia: A Systematic Review of Recovery and Safety Outcomes

**DOI:** 10.7759/cureus.116197

**Published:** 2026-09-13

**Authors:** Ajay Pesala, Thinless Angmo, Gokul Krishnan S, Sonik G Shah, Palak Shah, Shivali Pandey

**Affiliations:** 1 Department of Anaesthesia and Critical Care, Magna Mother and Child Hospital, Rajahmundry, IND; 2 Department of Anaesthesia, Atal Bihari Vajpayee Institute of Medical Sciences (ABVIMS) and Dr. Ram Manohar Lohia Hospital, New Delhi, IND; 3 Department of Anaesthesia, Kasturba Medical College, Manipal, IND; 4 Department of Anaesthesia, Dr. N.D. Desai Faculty of Medical Science and Research Centre, Dharmsinh Desai University, Nadiad, IND; 5 Department of Pharmacology, Smt. B. K. Shah Medical Institute and Research Centre, Sumandeep Vidyapeeth, Vadodara, IND; 6 Department of Anaesthesia, Maharani Laxmi Bai Medical College, Jhansi, IND

**Keywords:** acceleromyography, electromyography, general anesthesia, neuromuscular monitoring, residual blockade

## Abstract

Quantitative neuromuscular monitoring is increasingly used during general anesthesia to confirm recovery after neuromuscular blocking agents (NMBAs) and reduce postoperative respiratory risk. Residual neuromuscular blockade remains a preventable concern, yet monitoring practices, device performance, reversal use, and recovery thresholds remain variable across perioperative settings. This systematic review evaluated evidence on quantitative neuromuscular monitoring in general anesthesia, focusing on recovery assessment, residual blockade detection, device agreement, reversal practice, and postoperative safety outcomes. Literature was searched in PubMed/MEDLINE, Scopus, Google Scholar, and Web of Science. After duplicate removal, screening, and eligibility assessment, 10 studies were included for qualitative synthesis. Data were extracted on study design, sample size, monitoring modality, recovery thresholds, respiratory events, and key findings. Risk of bias was assessed using the Cochrane Risk of Bias 2 tool (RoB 2) for randomized studies and the Risk of Bias in Non-randomized Studies of Interventions (ROBINS-I) for non-randomized studies. The included studies showed that train-of-four (TOF)-based assessment was central to identifying incomplete recovery, commonly using a TOF ratio of at least 0.90. Residual blockade was associated with oxygen requirement, hypoxemia, critical respiratory events, and pulmonary complications. Device comparisons showed that acceleromyography, electromyography, TOF-Cuff, TOF-Scan, and ultrasonography were not always interchangeable. Routine quantitative monitoring with correct interpretation may improve extubation safety and postoperative recovery.

## Introduction and background

Neuromuscular blocking agents (NMBAs) are routinely administered during general anesthesia to facilitate tracheal intubation, optimize surgical conditions, and prevent unwanted patient movement [1]. Incomplete recovery from neuromuscular blockade may persist despite apparent clinical recovery or pharmacological reversal and is referred to as residual neuromuscular blockade (RNMB) [1]. RNMB is clinically important because it has been associated with impaired upper-airway function, ventilatory dysfunction, hypoxemia, postoperative pulmonary complications, delayed recovery, and other adverse respiratory events [1]. Reliable confirmation of neuromuscular recovery before extubation is therefore an important component of perioperative safety.

Quantitative neuromuscular monitoring provides an objective numerical assessment of neuromuscular transmission, most commonly through measurement of the train-of-four (TOF) ratio [2]. In contrast to clinical tests such as head lift or hand grip and qualitative visual or tactile assessment of muscle responses, quantitative monitoring can detect degrees of residual weakness that may not be apparent clinically [2]. A TOF ratio of ≥0.90 at the adductor pollicis is widely used as the threshold indicating adequate neuromuscular recovery before extubation [3]. Accurate interpretation nevertheless depends on appropriate stimulation patterns, electrode and device positioning, monitoring site, calibration, and, for some technologies, normalization of baseline measurements [3,4].

Quantitative monitoring can also guide reversal after rocuronium-induced blockade, with sugammadex or neostigmine selected according to block depth and objective TOF findings [5]. Routine implementation remains inconsistent and may be influenced by clinician education, institutional protocols, workflow integration, documentation, and device availability [6]. Advances in neuromuscular monitoring continue to refine objective assessment and device application in perioperative practice [7]. Quantitative monitoring remains clinically relevant even with the availability of sugammadex, as pharmacological reversal alone does not confirm complete neuromuscular recovery [8,9]. Evidence-based neuromuscular block management therefore emphasizes objective assessment of recovery alongside appropriate reversal strategies [10]. Variability among national and international recommendations and between monitoring technologies further supports the need for systematic evaluation of current evidence [11]. Quantitative monitoring has also shown feasibility across different perioperative populations [12], may influence rocuronium use and neuromuscular-block management [13], and newer quantitative approaches such as pupillometry continue to be explored [14].

Objective of the review

Using a PICO-oriented framework, this systematic review evaluates patients undergoing general anesthesia with neuromuscular blockade (Population), quantitative neuromuscular monitoring and objectively guided recovery assessment (Intervention/Exposure), alternative monitoring methods, usual clinical assessment, or different quantitative devices where reported (Comparator), and TOF recovery, residual neuromuscular blockade, reversal practices, device agreement, hypoxemia, oxygen requirement, pulmonary complications, and critical respiratory events (Outcomes). The objective is to determine the clinical contribution of quantitative neuromuscular monitoring to recovery assessment, reversal and extubation decisions, device performance evaluation, and postoperative respiratory safety.

## Review

Methodology

Study Design

This systematic review evaluated the role of quantitative neuromuscular monitoring during general anesthesia, with particular emphasis on neuromuscular recovery, residual neuromuscular blockade, monitoring-device performance, reversal practices, and postoperative respiratory safety. Eligible clinical studies included those assessing TOF monitoring, residual neuromuscular blockade, device agreement, reversal strategies, and respiratory complications. Owing to substantial clinical and methodological heterogeneity across study designs, monitoring technologies, surgical populations, recovery definitions, anatomical monitoring sites, and outcome-assessment time points, a quantitative meta-analysis was considered inappropriate. A qualitative synthesis was therefore undertaken. The review followed a structured systematic-review process comprising literature identification, duplicate removal, title and abstract screening, full-text eligibility assessment, data extraction, risk-of-bias evaluation, and qualitative synthesis, with reporting aligned to the PRISMA (Preferred Reporting Items for Systematic Reviews and Meta-Analyses) 2020 framework [15].

Search Strategy

A structured literature search was conducted in PubMed/MEDLINE, Scopus, Web of Science, and Google Scholar during the first week of July 2026, with the final search completed on July 5, 2026. Search concepts were developed around quantitative neuromuscular monitoring, TOF assessment, residual neuromuscular blockade, reversal, recovery, extubation, and postoperative respiratory safety. Boolean operators (“AND” and “OR”) were used to combine controlled and free-text terms. Searches were restricted to English-language human studies. No publication-date restriction was applied during database searching. The database-specific search strings, limits applied, and number of records retrieved from each source are reported in Table 1 to ensure reproducibility of the literature search.

**Table 1:** Database-Specific Search Strategies and Records Retrieved TOF: train-of-four; TS: topic search

Database	Search Strategy	Limits/Filters	Records Retrieved
PubMed/MEDLINE	("neuromuscular monitoring" OR "quantitative neuromuscular monitoring" OR "train-of-four" OR "train of four" OR "TOF ratio") AND ("general anesthesia" OR "general anaesthesia") AND ("neuromuscular blockade" OR "residual neuromuscular blockade" OR "residual paralysis" OR recovery OR reversal OR extubation) AND (respiratory OR hypoxemia OR "pulmonary complications" OR safety)	Humans; English language	82
Scopus	TITLE-ABS-KEY(("neuromuscular monitoring" OR "quantitative neuromuscular monitoring" OR "train-of-four" OR "TOF ratio") AND ("general anesthesia" OR "general anaesthesia") AND ("neuromuscular blockade" OR "residual neuromuscular blockade" OR recovery OR reversal OR extubation) AND (respiratory OR hypoxemia OR "pulmonary complications" OR safety))	English language	49
Web of Science	TS=(("neuromuscular monitoring" OR "quantitative neuromuscular monitoring" OR "train-of-four" OR "TOF ratio") AND ("general anesthesia" OR "general anaesthesia") AND ("neuromuscular blockade" OR "residual neuromuscular blockade" OR recovery OR reversal OR extubation) AND (respiratory OR hypoxemia OR "pulmonary complications" OR safety))	English language	76
Google Scholar	Combinations of “quantitative neuromuscular monitoring”, “train-of-four”, “general anesthesia”, “residual neuromuscular blockade”, “reversal”, “extubation”, “hypoxemia”, and “postoperative respiratory complications”; results were screened sequentially in relevance-ranked order, with the first 45 unique records assessed for eligibility.	English-language clinical studies; results screened in relevance order	45
Total	—	—	252

Eligibility Criteria

Studies including quantitative neuromuscular monitoring or objective assessment of recovery after general anesthesia were included. Studies that could be included were randomized trials, prospective observational studies, retrospective cohort studies, and device-comparison studies. Studies were eligible if they reported at least one prespecified outcome, including TOF recovery, residual neuromuscular blockade, device agreement or accuracy, reversal-agent use, oxygen supplementation, hypoxemia, postoperative pulmonary complications, or critical respiratory events. In addition, studies that did not focus on general anesthesia, did not use neuromuscular monitoring, did not have outcome data, were not published in the English language, and were reviews, editorials, and commentaries were not considered in the review.

Study Selection

After duplicate removal, two reviewers independently screened titles and abstracts against the predefined eligibility criteria. Potentially eligible reports were assessed independently at the full-text stage. Disagreements were resolved through discussion and consensus, with adjudication by a third reviewer when required. Reasons for full-text exclusion were recorded, and the complete study-selection process was documented using the PRISMA 2020 flow framework.

Data Extraction

Data were independently extracted by two reviewers using a standardized data-extraction form. The extracted information was cross-checked for accuracy, and discrepancies were resolved through discussion and consensus; when agreement could not be reached, a third reviewer was consulted. Data collected from each included study comprised the author, publication year, study design, sample size, clinical setting, patient population, NMBA, monitoring device, comparator, recovery criteria, reversal-agent use, and reported safety outcomes. Key outcomes included residual neuromuscular blockade, TOF ratio recovery, device agreement, oxygen supplementation, hypoxemia, pulmonary complications, and critical respiratory events. All extracted data were checked for consistency before qualitative synthesis, with particular attention to clinically relevant recovery and postoperative safety outcomes.

Risk of Bias Assessment

Risk of bias was assessed independently by two reviewers using design-specific validated tools. Randomized and cluster-randomized trials were evaluated with RoB 2 and its appropriate extension, retaining all five original domains and the standard judgments of low risk, some concerns, or high risk [16]. Non-randomized studies were assessed with ROBINS-I across all seven original domains, using the categories low, moderate, serious, critical risk, or no information [17]. QUADAS-2 was reserved for studies with a true diagnostic-accuracy framework involving an index test and reference standard [18]. Device-comparison studies assessing agreement, correlation, onset, or recovery without an independent reference standard were instead evaluated according to their observational design. None of the included device-comparison studies met this diagnostic-accuracy framework; therefore, QUADAS-2 was not applied in the final assessment. Disagreements were resolved by consensus, with third-reviewer adjudication when required.

Data Synthesis

Owing to clinical and methodological heterogeneity among the included studies, a qualitative synthesis was performed rather than a meta-analysis. Heterogeneity was observed in study design, monitoring modality, anatomical monitoring site, surgical population, NMBA, recovery threshold, and outcome definition. Findings were synthesized according to quantitative neuromuscular recovery, residual neuromuscular blockade, device-comparison findings, reversal practices, and postoperative respiratory safety outcomes. Results were compared descriptively across studies, without pooled statistical estimates. Where reported by the original studies, event counts, proportions, effect estimates, 95% confidence intervals, and P-values were extracted and summarized descriptively without statistical pooling.

Results

Study Selection

The database search identified 252 records: 82 from PubMed/MEDLINE, 49 from Scopus, 76 from Web of Science, and 45 from Google Scholar. After removal of 41 duplicates, 211 records underwent title and abstract screening. Of these, 164 were excluded because they did not meet the predefined eligibility criteria. Forty-seven reports underwent full-text assessment, of which 37 were excluded because of insufficient relevant outcome data, failure to satisfy the eligibility criteria, or non-English publication. Ten studies were included in the final qualitative synthesis. The complete study-selection process is presented in the PRISMA flow diagram (Figure 1).

**Figure 1:**
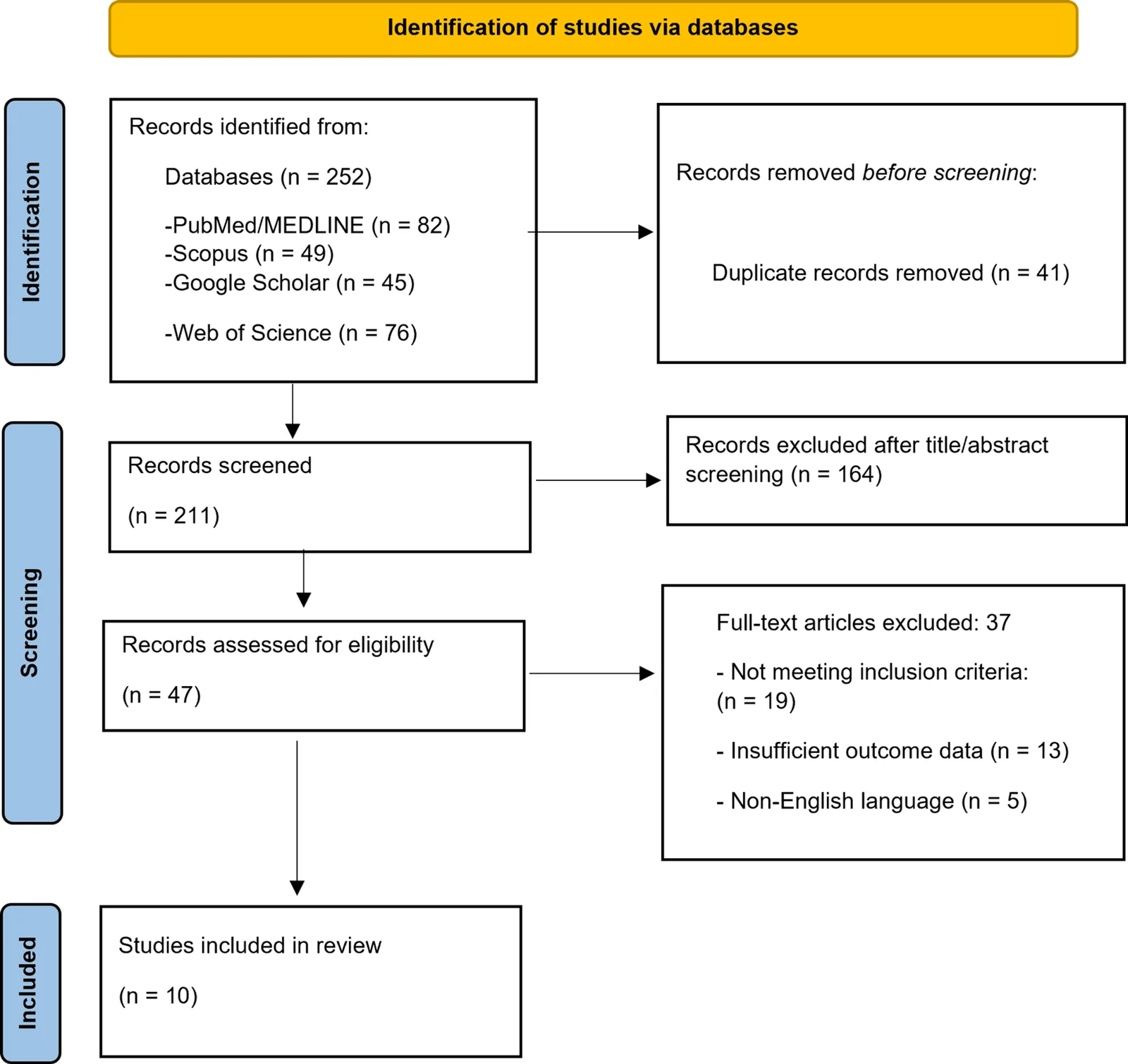
The PRISMA Flow Diagram PRISMA: Preferred Reporting Items for Systematic Reviews and Meta-Analyses

Study Characteristics

A total of 10 studies published between 2020 and 2026 were included, comprising randomized, prospective observational, retrospective cohort, and device-comparison designs. Sample sizes ranged from 25 participants to 14,592 surgical cases. Most studies evaluated adults undergoing general anesthesia with non-depolarizing NMBAs. Monitoring technologies included acceleromyography, electromyography, compressomyography, TOF-Cuff, TOF-Scan, IntelliVue neuromuscular transmission monitoring, TetraGraph, and diaphragm ultrasonography. Reported outcomes included residual neuromuscular blockade, TOF recovery, device agreement, reversal-agent use, supplemental oxygen requirement, hypoxemia, pulmonary complications, and critical respiratory events. Detailed study characteristics and principal findings are presented in Table 2.

**Table 2:** Characteristics and Key Findings of the Included Studies AMG: acceleromyography; NMBA: neuromuscular blocking agent; NMT: neuromuscular transmission; PACU: post-anesthesia care unit; RNMB: residual neuromuscular blockade; TOF: train-of-four; TOFR: train-of-four ratio

Study	Study Design	Sample/Setting	Monitoring Approach/Comparator	Main Recovery and Safety Outcomes	Key Finding
Díaz-Cambronero et al. [19]	Multicenter cluster-randomized crossover trial	2,314 adults undergoing general anesthesia in 34 Spanish centers	Educational intervention promoting quantitative neuromuscular monitoring, reversal when needed, and confirmation of TOF ratio ≥0.90	Postoperative residual curarization, quantitative monitoring use, reversal-agent use, postoperative pulmonary complications	The intervention increased quantitative monitoring use but did not significantly reduce postoperative residual curarization or increase reversal-agent use; postoperative pulmonary complications decreased.
Alenezi et al. [20]	Prospective cohort study	30 adult elective surgical patients in a tertiary hospital, Saudi Arabia	TOF-Scan acceleromyography in PACU; RNMB defined as TOF ratio <0.90	RNMB incidence and critical respiratory events in PACU	RNMB occurred in 53.3% of patients, and critical respiratory events were decreased among patients with RNMB than among those without RNMB.
Piersanti et al. [21]	Prospective observational study	90 adult patients admitted to the recovery room after NMBA exposure	Recovery-room acceleromyography; clinical NMBA management left to anesthesiologist discretion	RNMB incidence, oxygen supplementation, adverse respiratory events	RNMB occurred in 5% of patients; none of the patients with RNMB had received pharmacological reversal.
Feyling et al. [22]	Retrospective cohort study using anesthetic chart data	1,632 eligible cases from Oslo University Hospital, 2017–2020	Normalized vs. non-normalized acceleromyographic TOF ratios	Residual paralysis at extubation; bias between normalized and non-normalized TOF ratios	Residual paralysis was detected in 52.8% when a normalized TOF ratio <0.90 was applied; non-normalized acceleromyography overestimated recovery and could misclassify patients as adequately recovered.
Krijtenburg et al. [23]	Single-center retrospective cohort study	14,592 surgical cases from a large Dutch teaching hospital, 2015–2019	NMT monitoring trends before and after integrated automatic NMT recording; reversal-agent use assessed	Documented NMT monitoring, sugammadex use, extubation without documented TOF ratio ≥0.90	Automatic NMT recording coincided with increased documented monitoring and sugammadex use, but many patients still lacked documented TOF ratio ≥0.90 before extubation.
Markle et al. [24]	Prospective observational device-comparison study	50 patients; 25 non-obese and 25 obese	TOF-Cuff compared with TOF-Scan during atracurium-induced blockade	Time from NMBA administration to TOF ratio 0%; agreement between devices	TOF-Cuff and TOF-Scan showed clinically relevant intra-individual differences; the devices could not be used interchangeably, especially in obese patients.
Radkowski et al. [25]	Prospective observational clinical trial	25 adult patients receiving mivacurium	TOF-Cuff on upper arm compared with TOF-Scan on adductor pollicis	Onset time, recovery time, concordance between devices	TOF-Cuff and TOF-Scan showed good concordance for onset, but recovery correlation was weaker, and both devices showed false-negative results in patients with clinical signs of premature recovery.
Radkowski et al. [26]	Prospective observational clinical trial	36 adult elective surgical patients receiving mivacurium	TOF-Cuff on upper arm compared with TOF-Scan on corrugator supercilii/eyelid	Onset time, relaxation time, recovery time, false-negative monitoring results	TOF-Scan at the eyelid recorded a much shorter onset than TOF-Cuff; eyelid monitoring may be an alternative only with correction, and both methods showed false-negative findings.
Huang et al. [27]	Prospective randomized parallel-group clinical study	127 analyzed patients undergoing thyroid cancer surgery	Combined diaphragm ultrasonography + AMG vs. AMG alone vs. usual clinical practice	RNMB incidence and hypoxemia after extubation	Usual clinical practice without objective monitoring had higher RNMB and hypoxemia; diaphragm ultrasonography reduced RNMB and was non-inferior to AMG.
Danielsons et al. [28]	Prospective controlled observational device-comparison study	50 patients undergoing general anesthesia with atracurium	TetraGraph electromyography compared with IntelliVue acceleromyography	Time to TOF ratio ≥90%; time to TOF count 0; device agreement	TetraGraph recorded onset and recovery later than IntelliVue; IntelliVue reached TOF ratio ≥90% about 8.9 min earlier, suggesting acceleromyography may overestimate recovery and affect extubation timing.

Risk of Bias Assessment

Risk of bias was reassessed using the complete original domain structure and judgment categories appropriate to each study design. Standard RoB 2 was applied to individually randomized studies, the RoB 2 cluster-randomized extension was applied to the cluster-randomized trial, and ROBINS-I was applied to non-randomized studies using all seven domains. No domains were combined, relabeled, or condensed. Device-comparison studies without a diagnostic-accuracy reference-standard framework were assessed according to their observational design. The complete domain-level and overall judgments are presented in Table 3.

**Table 3:** Risk of Bias Assessment of the Included Studies Using Design-Specific Assessment Tools RoB 2 judgments are reported using the categories low risk, some concerns, and high risk. ROBINS-I judgments are reported using the categories low, moderate, serious, critical risk, or no information. All original domains of the respective assessment tools are reported separately, without combining or relabeling domains. RoB 2: Risk of Bias 2; ROBINS-I: Risk of Bias in Non-randomized Studies of Interventions

Study	Assessment Tool	Domain-Level Judgments	Overall Judgment
Díaz-Cambronero et al. [19]	RoB 2	Randomization process: Low; Deviations from intended interventions: Some concerns; Missing outcome data: Low; Measurement of the outcome: Low; Selection of the reported result: Low	Some concerns
Alenezi et al. [20]	ROBINS-I	Confounding: Moderate; Selection of participants: Moderate; Classification of interventions: Low; Deviations from intended interventions: Low; Missing data: Low; Measurement of outcomes: Low; Selection of reported result: Moderate	Moderate
Piersanti et al. [21]	ROBINS-I	Confounding: Moderate; Selection of participants: Low; Classification of interventions: Low; Deviations from intended interventions: Moderate; Missing data: Low; Measurement of outcomes: Low; Selection of reported result: Low	Moderate
Feyling et al. [22]	ROBINS-I	Confounding: Moderate; Selection of participants: Moderate; Classification of interventions: Low; Deviations from intended interventions: Low; Missing data: Low; Measurement of outcomes: Low; Selection of reported result: Moderate	Moderate
Krijtenburg et al. [23]	ROBINS-I	Confounding: Moderate; Selection of participants: Moderate; Classification of interventions: Low; Deviations from intended interventions: Moderate; Missing data: Low; Measurement of outcomes: Moderate; Selection of reported result: Moderate	Moderate
Markle et al. [24]	ROBINS-I	Confounding: Moderate; Selection of participants: Low; Classification of interventions: Low; Deviations from intended interventions: Low; Missing data: Low; Measurement of outcomes: Moderate; Selection of reported result: Low	Moderate
Radkowski et al. [25]	ROBINS-I	Confounding: Moderate; Selection of participants: Low; Classification of interventions: Low; Deviations from intended interventions: Low; Missing data: Low; Measurement of outcomes: Moderate; Selection of reported result: Low	Moderate
Radkowski et al. [26]	ROBINS-I	Confounding: Moderate; Selection of participants: Low; Classification of interventions: Low; Deviations from intended interventions: Low; Missing data: Low; Measurement of outcomes: Moderate; Selection of reported result: Low	Moderate
Huang et al. [27]	RoB 2	Randomization process: Low; Deviations from intended interventions: Some concerns; Missing outcome data: Low; Measurement of the outcome: Low; Selection of the reported result: Some concerns	Some concerns
Danielsons et al. [28]	ROBINS-I	Confounding: Moderate; Selection of participants: Low; Classification of interventions: Low; Deviations from intended interventions: Low; Missing data: Low; Measurement of outcomes: Moderate; Selection of reported result: Low	Moderate

Quantitative Neuromuscular Recovery

Across the included studies, adequate neuromuscular recovery was generally assessed using quantitative TOF measurements, with a TOF ratio ≥0.90 commonly used as the recovery threshold. Residual neuromuscular blockade was reported in 53.3% of 30 patients when defined as a TOF ratio <0.90 [20]. Another prospective study identified residual blockade in 5% of 90 patients evaluated in the recovery room [21]. A retrospective cohort reported residual paralysis in 52.8% of eligible cases when normalized acceleromyographic TOF ratios were applied, indicating that normalization influenced classification of recovery [22]. A cluster-randomized educational intervention increased the use of quantitative monitoring, although postoperative residual curarization was not significantly reduced [19]. Quantitative recovery findings are summarized in Figure 2.

**Figure 2:**
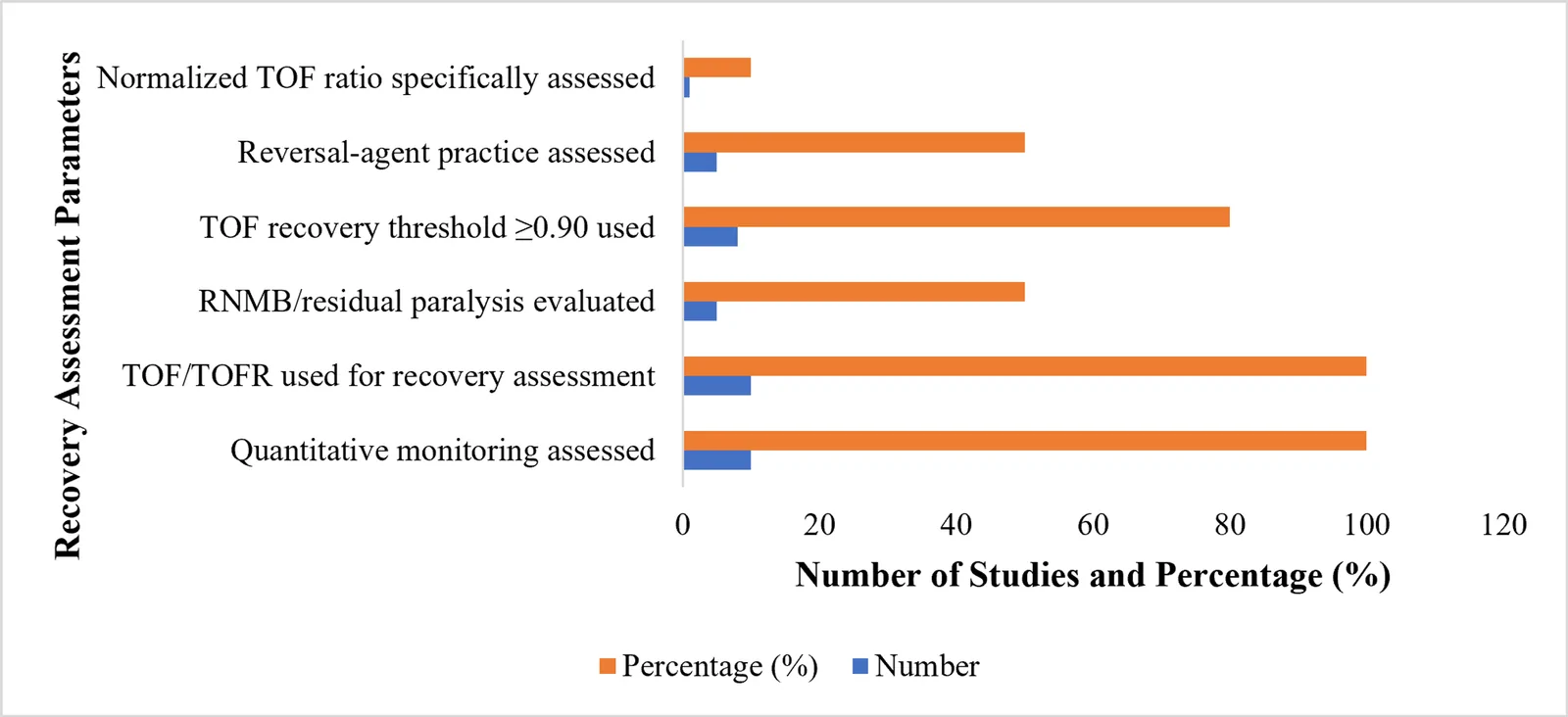
Quantitative Monitoring and Recovery Assessment Across Included Studies TOF: train-of-four; RNMB: residual neuromuscular blockade; TOFR: train-of-four ratio

Device-Comparison Findings

Device-comparison studies demonstrated differences in measured onset and recovery according to monitoring technology and anatomical site. Clinically relevant intra-individual differences were reported between TOF-Cuff and TOF-Scan, particularly in patients with obesity [24]. Good agreement was observed for onset measurements between TOF-Cuff and TOF-Scan, whereas agreement during recovery was weaker [25]. TOF-Scan monitoring at the eyelid produced shorter onset measurements than TOF-Cuff monitoring at the upper arm [26]. In another comparison, IntelliVue acceleromyography reached a TOF ratio ≥90% approximately 8.9 minutes earlier than TetraGraph electromyography [28]. The magnitude and direction of differences in onset and recovery measurements varied according to the monitoring device and anatomical site.

Postoperative Clinical Safety Outcomes

Postoperative respiratory outcomes were reported in a subset of the included studies. RNMB was identified in 16 of 30 patients (53.3%), and critical respiratory events occurred more frequently among patients with RNMB [20]. Another study reported RNMB in 5% of 90 recovery-room patients, with none of the affected patients having received pharmacological reversal [21]. In the randomized study, RNMB and post-extubation hypoxemia were more frequent with usual clinical practice than with objective monitoring [27]. The cluster-randomized intervention increased quantitative monitoring use but did not significantly reduce postoperative residual curarization; postoperative pulmonary complications were lower during the intervention condition [19]. Where reported by the original studies, event counts, proportions, effect estimates, 95% confidence intervals, and P-values were summarized descriptively without statistical pooling. Figure 3 summarizes the proportion of included studies reporting device-comparison findings and postoperative respiratory safety outcomes, including RNMB, oxygen supplementation or hypoxemia, pulmonary complications, and critical respiratory events.

**Figure 3:**
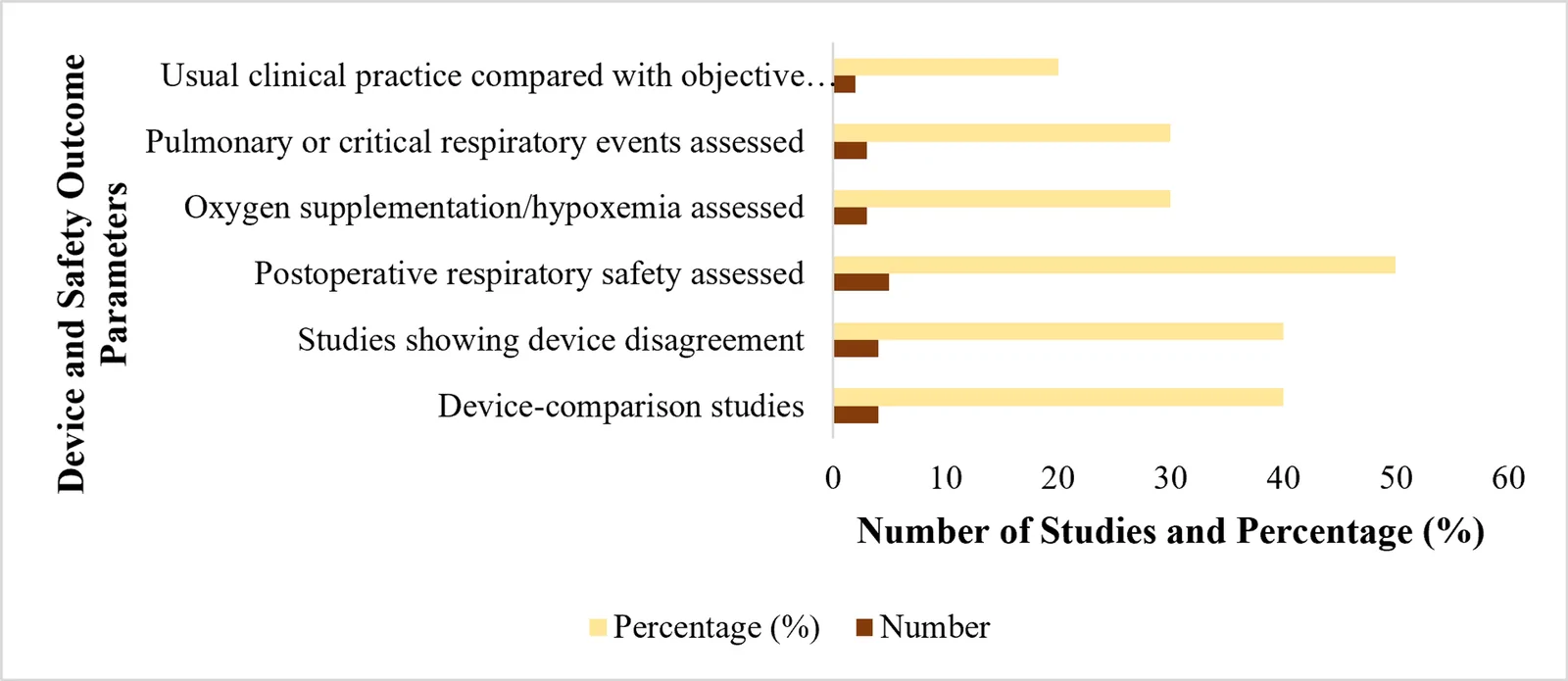
Summary of Device-Comparison Findings and Postoperative Respiratory Safety Outcomes

Discussion

This systematic review synthesized evidence from 10 studies evaluating quantitative neuromuscular recovery, residual neuromuscular blockade, monitoring-device performance, reversal practices, and postoperative respiratory outcomes. The principal finding was that residual neuromuscular blockade remained detectable across different perioperative settings despite the availability of pharmacological reversal and quantitative monitoring. TOF-based assessment, most commonly using a recovery threshold of ≥0.90, provided an objective measure of neuromuscular recovery and identified incomplete recovery that may not be reliably recognized using clinical assessment alone. The findings are consistent with contemporary guidance recommending quantitative assessment at the adductor pollicis and confirmation of adequate TOF recovery before extubation [5].

A TOF ratio of approximately 0.90 or greater was the most commonly applied threshold for adequate recovery among the included studies [29]. Residual blockade was associated in several studies with oxygen requirement, hypoxemia, critical respiratory events, and postoperative pulmonary complications [30]. These findings support an association between residual neuromuscular blockade and adverse postoperative respiratory outcomes; they do not establish that quantitative monitoring itself directly prevents these complications. The observed outcomes may also be influenced by the depth and duration of neuromuscular blockade, reversal strategy, patient characteristics, anesthetic technique, surgical procedure, and postoperative care [31]. Quantitative monitoring is most directly supported as a method for confirming recovery and informing reversal and extubation decisions.

Implementation remained inconsistent across the included evidence. Educational interventions and automated documentation systems increased recorded use of quantitative monitoring, but greater monitoring uptake did not uniformly eliminate residual blockade or incomplete documentation [19,23]. This suggests that implementation depends on more than device availability or education alone. Institutional protocols, standardized reversal pathways, documentation requirements, workflow integration, and audit-feedback mechanisms may be required to achieve consistent practice [6,32].

Device performance was another important source of variability. Comparisons involving TOF-Cuff, TOF-Scan, acceleromyography, electromyography, TetraGraph, IntelliVue NMT, and diaphragm ultrasonography demonstrated differences in onset and recovery measurements [24-28]. Agreement was particularly variable near recovery thresholds relevant to extubation. These findings indicate that measurements obtained from different technologies or anatomical sites should not automatically be considered interchangeable. Device-specific measurement principles, monitoring site, calibration, and known performance limitations should be considered when interpreting recovery [33].

Normalization was particularly relevant for acceleromyographic measurements. Non-normalized TOF ratios may overestimate neuromuscular recovery because baseline acceleromyographic ratios can exceed 1.0 [22,34]. Failure to account for this effect may result in misclassification of patients as adequately recovered when normalized values remain below the accepted recovery threshold. Accurate quantitative monitoring therefore depends not only on device availability but also on appropriate calibration, normalization, electrode placement, monitoring site, and threshold interpretation [34].

Diaphragm ultrasonography was identified as a potential adjunct to conventional quantitative neuromuscular monitoring. In the included randomized study, combined diaphragm ultrasonography and acceleromyography was associated with lower frequencies of residual neuromuscular blockade and hypoxemia than usual clinical practice without objective monitoring. These findings suggest that respiratory muscle assessment may provide additional information in selected clinical situations. Its routine role remains uncertain, and wider implementation would require standardized measurement thresholds, operator training, and further validation before it could be considered an alternative to established TOF-based quantitative monitoring [33,34].

Finally, based on the findings of this study, we could recommend the use of routine quantitative neuromuscular monitoring for patients in general anesthesia who are treated with non-depolarizing NMBAs. Objective monitoring allows verification of recovery and can inform reversal and extubation decisions; its independent effect on postoperative respiratory complications remains less certain. Additionally, evidence demonstrates that monitoring quality relies on the correct choice of devices, the correct interpretation, documentation, and incorporation into clinical workflow. Monitoring standards, confirmation of appropriate recovery before extubation, reversal of the action when indicated, and institutional reinforcement of monitoring standards are practical strategies that should be included in a practical safety plan.

Limitations and Future Directions

This review has a few limitations. Direct comparison among studies was restricted by heterogeneity in study design, sample size, monitoring technology, anatomical monitoring site, NMBA, reversal strategy, recovery threshold, and outcome definition. Several device-comparison studies included relatively small samples, limiting precision and generalizability. Retrospective studies were susceptible to incomplete documentation and confounding, and blinding was difficult in many perioperative monitoring studies. The predominance of observational evidence also limits causal inference regarding whether quantitative monitoring itself reduces postoperative respiratory complications. Owing to these clinical and methodological differences, quantitative meta-analysis was not considered appropriate.

Future studies should use standardized definitions of residual neuromuscular blockade, consistent TOF recovery thresholds, clearly defined postoperative respiratory outcomes, and prespecified monitoring protocols. Larger multicenter prospective studies are needed to compare acceleromyography, electromyography, compressomyography, and ultrasonography under standardized clinical conditions. Implementation studies should evaluate whether mandatory quantitative monitoring pathways, combined with protocolized reversal and documentation, reduce residual blockade, hypoxemia, pulmonary complications, and unplanned postoperative respiratory support.

## Conclusions

Quantitative neuromuscular monitoring provides an objective approach to assessing recovery after general anesthesia with NMBAs. The reviewed evidence indicates that residual neuromuscular blockade remains clinically relevant and may persist when recovery is incompletely documented or assessed using clinical signs alone. A TOF ratio of approximately 0.90 or greater was the most commonly used threshold for adequate recovery. Device type, anatomical monitoring site, calibration, and normalization influenced measurement and recovery interpretation, indicating that different monitoring technologies should not automatically be regarded as interchangeable. Residual neuromuscular blockade was associated with oxygen requirement, hypoxemia, critical respiratory events, and postoperative pulmonary complications in several studies, although the available evidence does not establish that monitoring alone directly prevents these outcomes. Quantitative monitoring should therefore be integrated with appropriate reversal strategies, accurate interpretation, complete documentation, and standardized perioperative protocols. Larger multicenter studies using consistent monitoring and outcome definitions are required to determine its effect on patient-centered postoperative outcomes.
